# Construction of Novel Yeast Strains from *Candida tropicalis* KBKTI 10.5.1 and *Saccharomyces cerevisiae* DBY1 to Improve the Performance of Ethanol Production Using Lignocellulosic Hydrolysate

**DOI:** 10.21315/tlsr2023.34.2.5

**Published:** 2023-07-21

**Authors:** Jamaluddin, Eny Ida Riyanti, Nisa Rachmania Mubarik, Edy Listanto

**Affiliations:** 1 Graduate School of IPB University, IPB University, Jl. Raya Dramaga, Kampus IPB Dramaga Bogor 16680 West Java, Indonesia; 2 National Research and Innovation Agency (BRIN), Jl. Tentara Pelajar No 3A, Bogor 16111, Indonesia; 3 Department of Biology, Faculty of Mathematics and Natural Science, IPB University, Jl. Raya Dramaga, Kampus IPB Dramaga, Bogor 16680 West Java, Indonesia

**Keywords:** Biofuels, Fermentation, Genetic Engineering, Lignocellulosic, Yeast

## Abstract

Increased consumption of xylose-glucose and yeast tolerance to lignocellulosic hydrolysate are the keys to the success of second-generation bioethanol production. *Candida tropicalis* KBKTI 10.5.1 is a new isolated strain that has the ability to ferment xylose. In contrast to *Saccharomyces cerevisiae* DBY1 which only can produce ethanol from glucose fermentation. The research objective is the application of the genome shuffling method to increase the performance of ethanol production using lignocellulosic hydrolysate. Mutants were selected on xylose and glucose substrates separately and using random amplified polymorphic DNA (RAPD) analysis. The ethanol production using lignocellulosic hydrolysate by parents and mutants was evaluated using a batch fermentation system. Concentrations of ethanol, residual sugars, and by-products such as glycerol, lactate and acetate were measured using HPLC machine equipped with Hiplex H for carbohydrate column and a refraction index detector (RID). Ethanol produced by Fcs1 and Fcs4 mutants on acid hydrolysate increased by 26.58% and 24.17% from parent DBY1, by 14.94% and 21.84% from parent KBKTI 10.5.1. In contrast to the increase in ethanol production on alkaline hydrolysate, Fcs1 and Fcs4 mutants only experienced an increase in ethanol production by 1.35% from the parent KBKTI 10.5.1. Ethanol productivity by Fcs1 and Fcs4 mutants on acid hydrolysate reached 0.042 g/L/h and 0.044 g/L/h. The recombination of the genomes of different yeast species resulted in novel yeast strains that improved resistance performance and ethanol production on lignocellulosic hydrolysates.

**Highlights**

- Novel strains of Fcs1 and Fcs4 were obtained with different xyl1 and pdc5 genes from the *Candida tropicalis* KBKTI 10.5.1 and *Saccharomyces cerevisiae* DBY1 using genome shuffling method.
- The xylose-glucose consumption of Fcs1 and Fcs4 mutant on lignocellulosic hydrolyzate increased compared to their parents.
- The ethanol productivity of the Fcs1 mutant increased by 16.67%–27.27%, while the Fcs4 mutant reached 22.22%–33.33% compared to the parental KBKTI 10.5.1 and DBY1 when using acid hydrolysate.

## INTRODUCTION

Renewable and environmentally friendly energy sources such as biofuels from biomass is essential strategies for substituting the use of fossil energy (Morais *et al*. 2018). The use of bioethanol has been proven to reduce carbon emissions compared to gasoline and diesel. The International Energy Agency (IEA) reports that the use of bioethanol is estimated to reduce carbon emissions by about 2.1 gigatons per year by 2050 if it is produced stably (Jin & Sutherland 2016). Bioethanol can be produced from abundant materials and can regenerate plants containing glucose, starch and lignocellulose. Bioethanol production from lignocellulosic is more desirable than glucose and starch because socioeconomically, sugar or starch leads to competition with food and feed needs (Meneses *et al*. 2017; Robak & Balcerek 2018).

Many factors affect the productivity of ethanol production using lignocellulosic, including the lack of ability of microorganisms to ferment xylose-glucose and low viable in lignocellulosic hydrolysate media (Bušić *et al*. 2018; Robak & Balcerek 2018). The bioethanol production from xylose-glucose still uses a separate reactor since its inability or slower for co-fermenting both xylose and glucose (Cardona & Sánchez 2007). In addition, hydrolysis proceses of lignocellulosic produce several inhibitor compounds such as furfural, HMF, vanillin, acetic acid and formic acid which can decrease yeast viability to ethanol production (Riyanti & Listanto 2017; Sjulander & Kikas 2020).

*C. tropicalis* KBKTI 10.5.1 1 can ferment xylose to produce ethanol, while *S. cerevisiae* DBY1 produce high ethanol concentration from glucose. Efforts to increase the ability to produce ethanol from xylose-glucose co-substrate continue to be carried out. Expression of xylose isomerase from *Burkholderia cenocepacia* to *S. cerevisiae* was performed (Vilela *et al*. 2013). However, the resulting strain still consumes xylose very slowly compared to glucose and produces relatively low ethanol productivity. Efforts to increase the consumption of xylose-glucose have also been carried out with an adaptive evolution approach on media containing xylose (Vilela *et al*. 2015). Research with this approach has produced a yeast strain that ferment xylose-glucose quickly and almost simultaneously. However, reports on genetic improvement of yeasts to increase ethanol fermentation of xylose-glucose co-substrates and their tolerance to lignocellulosic hydrolysates very limited. The complexity of the regulation system of co-fermenting xylose-glucose to produce ethanol and the response of yeast to inhibitor compounds in lignocellulosic hydrolysates makes it challenging to engineer its metabolism by inserting and/or knocking out several genes responsible for the regulation system, and time consuming. Therefore, undirected mutation technology through the genome shuffling method has the potential to be developed to obtain superior yeast strains for lignocellulosic hydrolysate fermentation.

Genome shuffling is a metabolic engineering method based on interprotoplast fusion. Protoplast fusion causes random recombination at the chromosomal level and can result in rapid multiplication of mutations so that the new strains formed have very diverse phenotypic properties (Biot-Pelletier & Martin 2014). Several studies have reported that genome shuffling can increase temperature and ethanol tolerance in *S. cerevisiae* 2013 (Orosco *et al*. 2017) as well as increase productivity and ethanol tolerance in *S. cerevisiae* and *Pichia stipites* (Jetti *et al*. 2018). Therefore, potential new strains could be obtained from *C. tropicalis* KBKTI 10.5.1 and *S. cerevisiae* DBY1 to increase xylose-glucose fermentation and have high tolerance to lignocellulosic hydrolysates.

## MATERIALS AND METHODS

### Yeast Strains

*C. tropicalis* KBKTI.10.5.1 and *S. cerevisiae* DBY1 were isolated from traditional Indonesian fermented foods that were carried out at the Molecular Biology Laboratory of the Indonesian Centre for Agricultural Biotechnology and Genetic Resources Research (Indonesia). KBKTI.10.5.1 isolate was able to produce 0.8 g/L ethanol from 20 g/L xylose, while DBY1 could produce 21.98 g/L ethanol from 50 g/L glucose for 96 h of incubation.

### Culture Preparation and Media Preparation

Yeast isolates were cultured on YPD agar (1% yeast extract, 2% peptone, 2% D-glucose, 2% agar) pH 5 for 24 h. Seed culture for fermentation were prepared on liquid YPD media and incubated overnight at 30ºC with 150 rpm agitation. Seed culture, as much as 10% (v/v) was used for fermentation. Lignocellulosic hydrolisate of elephant grass (*Pennisetum purpureum* Schum) was obtained from 10 g of lignocellulose dissolved in 50 mL of acid solution (3% H2SO4) and alkaline solution (3% NaOH) for 24 h followed by high temperature and pressure treatment using autoclave. The pH of the lignocellulosic hydrolysate was adjusted to 5 using HCL and NaOH solutions (Riyanti *et al*. 2019).

### Genome Shuffling (GS)

The construction of the novel strain was carried out by transferring DBY1 genomic DNA into KBKTI.10.5.1 cells using the genome shuffling method (Zhang & Geng 2012). The GS step was carried out with slight modifications to the pre-treatment solution using 0.1 M CaCl_2_. GS was carried out through electroporation technique using the MicroPulser^TM^ 165–2100 electroporation system. A total of 100 μL of GS results were spread into YPX media (1% yeast extract, 2% peptone, 2% d-xylose, 2% agar) pH 5 and incubated for 3 days at 30ºC. The growing colonies were purified and mutants were selected.

### Mutant Selection

Parent and mutants were inoculated into YPX and YPG agar (containing 1% yeast extract, 2% peptone, 2% D-xylose/D-glucose, 2% agar pH 5) and incubated overnight at 30ºC, 150 rpm. Cultures with 1 mL (OD_600_ = 0.5) were inoculated into 9 mL of YPX and YPG media and incubated for 24 h at 30ºC, 150 rpm. The ability of wild types and mutants to produce ethanol from xylose and glucose was evaluated by measuring the concentration of ethanol and the remaining fermented liquid substrate using HPLC (Riyanti *et al*. 2019). Mutant selection was also carried out through genome profile analysis using the Random Amplified Polymorphic DNA (RAPD) method with primers in Table 1.

### Evaluation of Cell Morphology and Genes Responsible for Ethanol Metabolism of Selected Strains

The selected strain cells were grown in YPD broth at 30ºC for 48 h. Cell cultures were prepared in a haemocytometer plate (depth 0–1 mm 1/400 mm^2^) and observed using a microscope. Several genes responsible for ethanol production and xylose utilisation such as *xyl*1, *adh*1, *xks*1, *pdc*1, *pdc*5, *pdc*6 were confirmed qualitatively using Polymerase Chain Reaction (PCR) method. The primers used are presented in Table 2.

### Ethanol Fermentation, Hydrolysed Sugar Analysis, Analysis of Fermentation Results

Parent and mutants were grown in YPXG broth media (1% yeast extract, 2% peptone, 2% D-xylose, 5% D-glucose) and elephant grass (*Pennisetum purpureum* Schum) lignocellulosic hydrolysate medium (pH 5). Fermentation was carried out for 72 h on YPXG media and 24 h on lignocellulosic hydrolysate media at 30ºC, 150 rpm. Observations were made every 24 h on YPXG media and 4 h, 8 h and 24 h on lignocellulosic hydrolysate media. The content of xylose-glucose resulted from lignocellulosic hydrolysate and its products such as ethanol, glycerol, lactate and acetate were analysed using HPLC Agilent Technologies 1260 Infinity with a Hi-plex H for carbohydrate column and a refraction index detector (RID) detector (Riyanti *et al*. 2019).

### Kinetic Parameters and Data Analysis

Cell biomass was obtained from the conversion of the OD_600_ value (Myers *et al*. 2013). The ability of parental and mutant yeasts to consume xylose-glucose was calculated using the following formula:


$$\text{Percentage of xylose}-\text{glucose consumption}=\frac{S0-S}{S0}\times 100\%$$

where *S*0 = initial xylose-glucose concentration and *S* = xylose-glucose concentration after fermentation.

The results of each concentration of ethanol and cell biomass obtained were then used to calculate the kinetic parameters of ethanol production. The kinetic parameters of ethanol production include ethanol yield (Yp/s), biomass yield (Yx/s) and ethanol productivity (Qp) (Moremi *et al*. 2020).


$$\begin{array}{l} {\text{Y}}_{\text{p}/\text{s}}=\frac{P}{\Delta S}\\ {\text{Y}}_{\text{x}/\text{s}}=\frac{X}{\Delta S}\\ \text{Qp}=\frac{P}{\text{Fermentation time}} \end{array}$$

where *P =* fermented products, *X* = cell biomass and ΔS = concentration of consumed substrate.

The RAPD data obtained is based on the amplification band scoring with a classification of “1” if there is an amplified band and “0” if there is no amplified band. The data were then analysed using the NTSYS-pc programme (Numerical Taxonomy and Multivariate Analysis System, Version 2.02i). All ethanol production performance data were analysed by simple mathematics using the Excel programme.

## RESULTS

### Selection of Potential Mutants for Ethanol Production from Xylose and Glucose

KBKTI 10.5.1 and DBY1 are isolates that have been identified using ITS1–ITS4 primers and have identified genomic characteristics using eight RAPD primers (Jamaluddin *et al*. 2021). RAPD is a DNA polymorphism produced by amplification of random DNA segments with a single primer sequence. The use of RAPD can be applied to distinguish between two individual organisms in one species (Suharsono *et al*. 2020). Thus, the approach to selecting potential fusants was to measure the fermentability of xylose and glucose into ethanol and by using RAPD technology.

**Figure 1:**
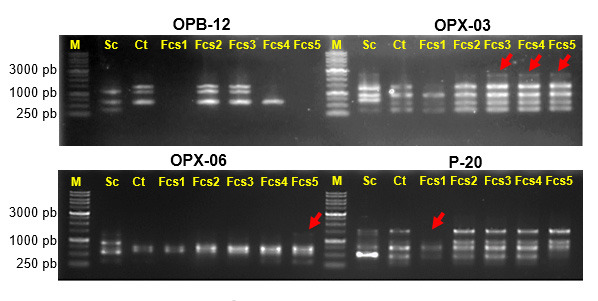
RAPD electrophorogram of parental and mutant yeast genomes on 1% agarose. (M) 1 kb Ladder DNA, (Sc) *S. cerevisiae* DBY1, (Ct) *C. tropicalis* KBKTI 10-5.1, (Fcs) mutant strains.

The construction of new strains using genome suffling by transferring genomic DNA of DBY1 into KBKTI 10.5.1 cell produced mutants which increased in xylose consumption and ethanol production (Table 3). In the screening process using glucose and xylose substrates, variable sugar uptake and ethanol production were observed from the GS mutants. Mutant Fcs2 experienced a decrease in xylose consumption rate compared to the parent of KBKTI 10.5.1. While mutants Fcs1, Fcs3 and Fcs5 shows an increase in xylose consumption rate and Fcs4 mutant show an increase in ethanol production using xylose and glucose substrate (Table 3). This phenomenon illustrates that there is a change in the genetic component which changes in metabolism. This is supported by the different genomic profiles between parents and mutants based on RAPD analysis (Fig. 1).

**Figure 2:**
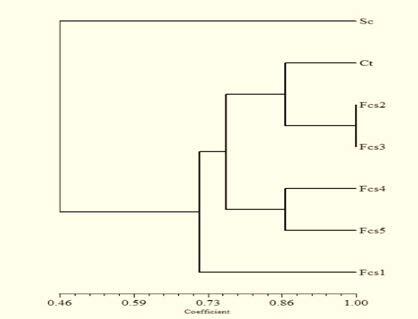
Cluster dendogram of parental and mutant yeast strains constructed with SAHN-UPGMA. (Sc) *S. cerevisiae* DBY1; (Ct) *C. tropicalis* KBKTI 10-5.1 and (Fcs) mutant strains.

The RAPD profile in Fig. 1 illustrates that GS causes the transfer or substitution of nucleotide bases from DNA DBY1 to genomic DNA of KBKTI 10.5.1. The existence of insertion or substitution of nucleotide bases causes the RAPD primers used to attach or not during the PCR process so that polymorphisms are formed. The results of the RAPD analysis showed that there was random recombination between the genome of DBY1 and KBKTI 10.5.1. The RAPD profile of parental and mutant polymorphisms using the OPX-03 primer showed that the mutants similar to the KBKTI 10.5.1, but there was an additional DNA band at 2500 bp as shown in the DBY1 profile. RAPD profiles with P-20 primer were found different between parent and mutans as band around 150 bp was do not detected in Fcs1, also band around 250 pb was not detected in Fcs 5. The difference in RAPD amplicon profiles as shown in Fig. 1 indicates the presence of genetic diversity in the constructed mutants. The resulting mutants were clustered in one cluster with the parent KBKTI 10.5.1. Fcs1 mutants were far apart to form separate sub-clusters at a genetic distance of 0.70. In addition, the Fcs4 and Fcs5 mutants grouped together to form a separate sub-cluster from the parent of KBKTI 10.5.1 (Fig. 2). The existence of genetic diversity produced can be an opportunity to produce superior strains. Fcs1 and Fcs4 mutants were then selected for further observation. Selection of Fcs1 and Fcs4 mutants based on the best ethanol production and xylose consumption compared to their parents.

Genomic DNA of *C. tropicalis* KBKTI 10.5.1 has also been tried to be transferred into *S. cerevisiae* DBY1 cells, but the yeast strain obtained was still unable to produce ethanol from xylose (unpublished data). The failure of the construction of the new yeast strain for ethanol production from xylose using of strategy the KBKTI 10.5.1 genomic DNA transfer into DBY1 cells was thought to be because *S. cerevisiae* DBY1 does not have a xylose reductase gene sequence, a gene that plays a role in catalysing xylose to enter ethanol metabolism (Mouro *et al*. 2020; Olofsson *et al*. 2011). Thus, the DNA sequence of the KBKTI 10.5.1 xylose reductase gene was not successfully inserted into DBY1 DNA because there was no homology between the two sequences.

### Evaluation of Cell Morphology and Genes Responsible for Ethanol Metabolism of Selected Mutant

**Figure 3:**
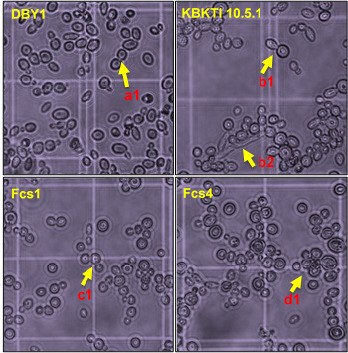
Morphology of parental and mutant yeast cells after 48 h of incubation on YPD media. (a1, b1, c1 and d1) budding, (b2) pseudohyphae.

The novel yeast strains that were constructed had morphological characters of cells which were thought to be inherited from the two parents. The two mutant cells were spherical in shape more closely resembled parental KBKTI 10.5.1 compared to the slightly oval shape of DBY1 cells. However, the mutant cells did not form pseudohyphae like the parent of KBKTI 10.5.1 which had been incubated at 30ºC for 48 h simultaneously with the two parents (Fig. 3).

**Figure 4:**
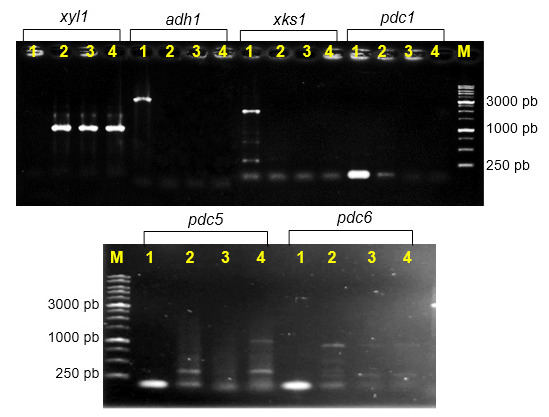
Electrophorogram of genes that play a role in ethanol metabolism. (M) 1 kb Ladder DNA; (1) *S. cerevisiae* DBY1; (2) *C. tropicalis* KBKTI 10-5.1; (3) Fcs1; and (4) Fcs4.

**Figure 5:**
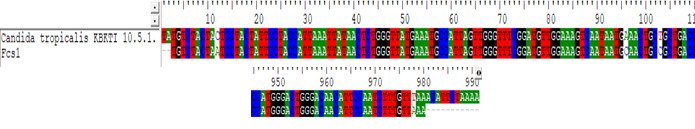
The results of the alignment analysis of the xylose reductase (xyl1) sequences of *C. tropicalis* KBKTI 10-5.1 and Fcs1 using the BioEdit programme.

In addition to cell morphology observations, several genes that play a role in ethanol metabolism were also evaluated qualitatively. Fig. 4 shows that KBKTI 10.5.1 and both mutants have the same amplicon xylose reductase profile, which was around 1,000 bp. However, the results of the sequence alignment analysis showed that there were four nucleotide base differences (Fig. 5). This indicated that the transfer of DBY1 DNA into KBKTI 10.5.1 cells caused a change in the nucleotide base arrangement in the KBKTI 10.5.1 genome. The results of the analysis of sequence homology in the *BLAST-n* programme showed that the xylose reductase KBKTI 10.5.1 was identical to the xylose reductase *Candida tropicalis* isolate GRA1 (99.59%) while the Fcs1 mutant changed to 99.28% against the xylose reductase isolate GRA1 gene. *C. tropicalis* isolate GRA1 is a comparison isolate, which has the gene sequences encoding xylose reductase available in the genebank (MF143598.1). Isolate GRA1 is able to reduce xylose to xylitol, which will enter the ethanol fermentation pathway, because it has a xylose reductase gene sequence (Ariyan & Uthandi 2019). The difference in the percentage similarity of the xylose reductase sequences of KBKTI 10.5.1 and Fcs1 isolates to GRA1 can be one of the benchmarks for the success of the genome shuffling that has been carried out. Changes in the arrangement of four nucleotide bases can change the amino acid composition of the mutant, this is indicated by the results of *BLAST-p* xylose reductase KBKTI 10.5.1 which has a homology percentage of 96.96% and Fcs1 xylose reductase reaches 96.27% against D-xylose reductase *C. tropicalis* (QED90344 .1).

**Figure 6:**
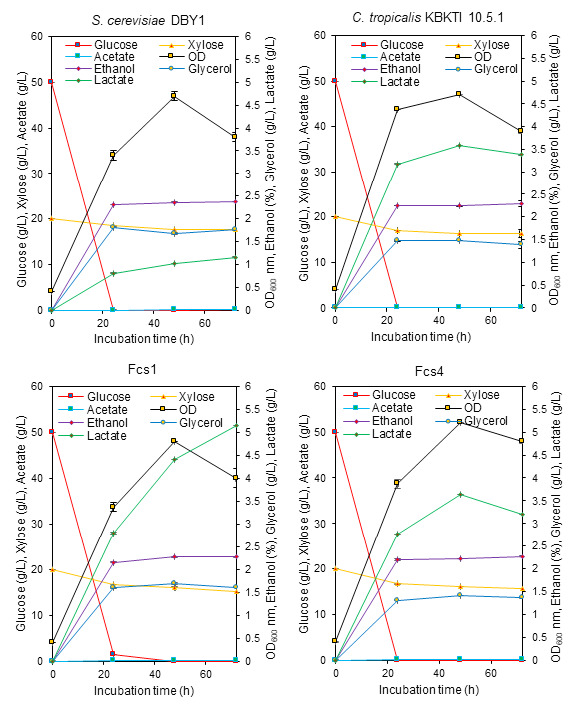
The bioethanol production performance of parental and mutant yeast in YPXG media.

The gene encoding pyruvate decarboxylase were also different. The results of the study in Fig. 4 show that there are several bands generated from the *pdc*5 and *pdc*6 PCR primers. This shows that the primer used is not yet specific. However, it can be seen that with *pdc*5 and *pdc*6 primers, Fcs1 and Fcs4 mutants are different from their parents. Differences in the amlicone profile of the gene encoding pyruvate decarboxylase may support differences in the production of by-products (lactate, acetate and glycerol), as shown in Fig. 6.

### Performance of Wild Type and Mutant Yeast for Ethanol Production on YPXG Media and Elephant Grass (*Pennisetum purpureum* Schum) Lignocellulosic Hydrolysate

Yeast strains capable of fermenting xylose-glucose to produce high ethanol concentrations are one of the keys to the success of ethanol production using lignocellulosic (Zabed *et al*. 2016). Our results showed that the KBKTI 10.5.1 strain as well as the two constructed mutants had the ability to produce approximately 2% ethanol from xylose-glucose fermentation for 72 h (Fig. 6). However, the ability to use xylose-glucose between parents and mutants was different. The DBY1, KBKTI 10.5.1 and the Fcs4 mutant were not able to use xylose-glucose simultaneously, in contrast to the Fcs1 mutant which was able to use xylose when glucose was still available in the medium. In addition, the consumption of xylose by the Fcs1 mutant was slightly higher than that of the two parents, but there was no increase in ethanol production. This phenomenon is thought to occur because the consumed xylose was converted to lactate, this can be seen from the increase in lactate production as the xylose concentration in the media decreases (Fig. 6). However, Fcs1 and Fcs4 mutants are potential to be used as second generation bioethanol production agents with further research on process engineering to minimise the formation of side products such as lactate, acetate and glycerol.

**Figure 7:**
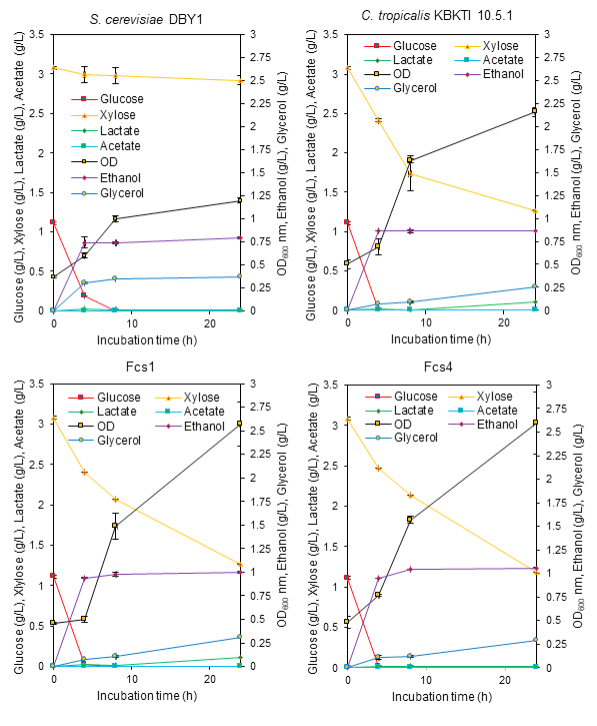
The bioethanol production performance of parental and mutant yeast in elephant grass lignocellulosic acid hydrolysate media.

**Figure 8:**
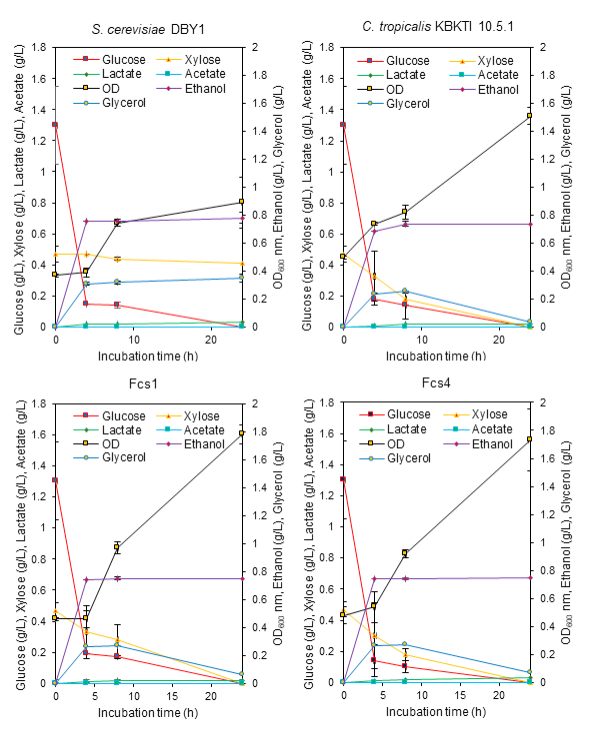
The bioethanol production performance of parental and mutant yeast in elephant grass lignocellulosic alkaline hydrolysate media.

The results of the study as shown in Fig. 6 can be seen that the use of xylose at a mixed concentration of 20 g/L–50 g/L xylose-glucose has not been able to significantly increase ethanol production. The research report showed that *Pichia kudriavzevii* and *C. tropicalis* experienced an increase of ethanol production at a 1:1 xylose-glucose mixture (Nweze *et al*. 2019). Therefore, it is necessary to optimise the concentration of the xylose-glucose mixture to increase ethanol production. In addition to substrate concentration, the ability to produce ethanol using lignocellulosic is also influenced by the presence of inhibitors produced in the hydrolysis process (Faizal *et al*. 2020; Chaudhary *et al*. 2021). Therefore, this study also reports the performance of ethanol by parental and mutant strains in lignocellulosic hydrolysate media presented as in Figs. 7 and 8.

Elephant grass (*Pennisetum purpureum* Schum) is one of the potential raw materials for bioethanol production (Vidal *et al*. 2017). Elephant grass biomass contains 60.20% cellulose, 23.80% hemicellulose (Minmunin *et al*. 2015), which can be hydrolysed into xylose and glucose. However, the concentration of sugar (xylose-glucose) obtained from the hydrolysis of acids and bases in this study was very low, reaching only 4.18 g/L and 1.77 g/L (Figs. 7 and 8). This can indicate that the hydrolysis of elephant grass biomass using acids and bases combined with pressure has not been maximised to increase the concentration of glucose and xylose. Sugar concentration below 10 g/L in bioethanol fermentation is not recommended because it can produce very little ethanol (Faizal *et al*. 2020). However, the results of our study need to be reported because through the production of bioethanol using lignocellulosic hydrolysates, the information on the ability to consume xylose-glucose and the growth ability of yeast strains that we obtained on the hydrolysate can be known.

The performance of xylose-glucose consumption between parents and mutants on both acid and alkaline hydrolysate media showed that the two mutants were more similar to the parents of KBKTI 10.5.1 (Figs. 7 and 8). The ability to consume glucose by DBY1 on acid hydrolysate media seemed to slow down, where at 4 h glucose was still available compared to the other three strains that had been used up. This is one of the effects of the concentration of ethanol produced being lower than the parent KBKTI 10.5.1 and the two mutants (Fig. 7). The results in Fig. 7 also show that at the concentration of the xylose-glucose mixture of about 3:1, the parent of KBKTI 10.5.1 and both mutants were able to use xylose-glucose simultaneously. This is indicated by the significant use of xylose at 4 h (Fig. 7). In addition, the ability to grow mutants on acid hydrolysate media was better when compared to the two parents, this was indicated by the high OD_600_ value and a slight increase in ethanol production (Fig. 7).

### Productivity of Parent and Mutant Yeast on Lignocellulosic Hydrolysate Media

The ability of bioethanol production by the parents and the resulting new strains are different. This difference can also be seen from the kinetic parameters as shown in Table 4. The ethanol productivity of the parents on YPXG media with a composition of 20 g/L-50 g/L xylose was better than that of the mutant, but on acid hydrolysate the ethanol productivity of the new strain was better. This can indicate that genomic recombination between the two parents resulted in improved productivity of resistance to lignocellulosic hydrolysates. The conversion of xylose-glucose to ethanol by strains Fcs1 and Fcs4 on acid hydrolysate media was also quite efficient. This can be seen from the product yield which reached 0.37 g/g. The xylose-glucose conversion efficiency of strains Fcs1 and Fcs4 was almost equivalent to the conversion of hexose with the addition of a lignocellulosic inhibitor by Saccharomyces cerevisieae isolate ISO12, which was 0.38 g/g, in which the ISO12 strain was claimed to be one of the superior candidates for the production of second-generation bioethanol (Wallace-Salinas & Gorwa-Grauslund 2013; Riyanti *et al*. 2019).

## DISCUSSION

Genetic mutation is one of the phenomena that can cause the diversity of organisms. The use of the concept of mutation has developed rapidly, such as to assemble a superior organism (Galhardo *et al*. 2007; Loewe & Hill 2010). This study uses a random mutation approach in two different species with the help of electric shocks. The use of electric shocks aims to enlarge the pores of the cell membrane so as to increase the permeability of the membrane so that DNA molecules can enter the cell (Nesin *et al*. 2011). The electric shock will cause an unused break in the second molecule of the parent yeast genomic DNA. This disconnection provides an opportunity for mutations to occur when there is a homologous wet tide and an incorrect reunification. Mutations that occur can reach more than one gene so that the mutants obtained have diversity at the genetic level (Figs. 1 and 2).

The constructed novel strain has a different physiology from the two wild types. Among the different physiological properties are the ability to produce ethanol from xylose, glucose and cause differences in cell morphology such as the formation of pseudohyphae. Pseudohyphae are formed from shoot cells, such as blastospores, which multiply, but the daughter cells do not separate from the parent cell and continue to elongate to resemble hyphae, so that there is a septum between the blastospore and the growing part of the cell, and in this section, there is a narrowed part (Veses & Gow 2009). The formation of pseudohyphae is closely related to the physiological properties of cells which are influenced by several factors such as nitrogen deficiency (Lackey *et al*. 2013; González *et al*. 2018), CO2 exposure (Sasani *et al*. 2016), high phosphate and growth period (Veses & Gow 2009), growing temperature conditions (Nadeem *et al*. 2013). The results of cell morphology observations as shown in Fig. 3 can illustrate that the physiological roles of parent and mutant in responding to environmental conditions (nutrients in YPD media, temperature and incubation time) were different. The difference in phenotype of an organism is part of the influence of its genetic expression (Young *et al*. 2019). Therefore, the transfer of DBY1 DNA to KBKTI 10.5.1 cells was suggested to cause the insertion of genetic material into the KBKTI 10.5.1 genome, thereby causing changes in some genetic expressions.

The novel strain that was constructed experienced an increase in the ability to consume xylose and produce ethanol. Yeast strains capable of fermenting xylose cannot use xylose until the glucose concentration is completely depleted. This can be explained by several factors, including because glucose can suppress the expression of genes responsible for xylose metabolism through the transcription factor Mig1. Cells that are in high glucose concentrations cause Mig1 to move from the cytoplasm to the nucleus and bind to gene promoters that can cause repression of xylose catabolism genes. After the glucose concentration in the medium decreases, the transcription factor Mig1 is transported back to the cytoplasm so that the repression is released (Rolland *et al*. 2002). This is one of the factors that slows down ethanol production in the presence of xylose in the media. High glucose concentration in the xylose-glucose mixture is one of strategy to increase ethanol production. This can be seen from the ability of the parent and mutant for 24 h to produce ethanol around 6.94 g/L–7.34 g/L from the fermentation of 20 g/L glucose (Table 3), compared to a mixture of 20 g/L–50 g/L xylose-glucose already can produce approximately 2% ethanol or about 17 g/L–18 g/L (Fig. 6). Moreover, the study results need to be reported because the constructed mutants have an increased ability to consume xylose (Table 3) and can even be used in together with glucose (Figs. 6, 7 and 8).

The use of xylose-glucose by KBKTI 10-5.1 and the two mutants on elephant grass lignocellulosic hydrolysate media showed that both were used simultaneously but were not able to maximally increase ethanol production (Figs. 7 and 8). This is thought to be caused by the very low concentration of total sugars, so that the sugars consumed is mainly used up for cell growth. In addition, there is no inhibitors separation process was done prior the fermentation process, therefore complex inhibitors produce during acid and alkaline hydrolysis may adding the stress condition during fermentation process.

Yeast cells are the main engine in fermentation, having the potential to be exposed to various types of fermentation stresses such as osmotic stress, temperature stress, ethanol stress and the presence of toxic compounds during the fermentation process (Burphan *et al*. 2018; Mukherjee *et al*. 2014). The resistance of yeast cells in responding to fermentation stress is an important factor that must be possessed by a fermentation agent (Riyanti & Listanto 2021). There is a direct relationship between fermentation efficiency and yeast resistance to stress which refers to the ability of yeast lines to adapt efficiently to unfavourable growing conditions (Saini *et al*. 2017). Lignocellulosic hydrolysate can produce several mixtures of inhibitor compounds that can inhibit ethanol production, cell growth, and enzyme biochemical activity (Palmqvist & Hahn-Hagerdal 2000). Therefore, OD_600_ nm in Figs. 7 and 8, showed that the newly obtained lines experienced faster growth in the lignocellulosic hydrolysate than the parent. This can represent the ability of tolerance and good productivity to the presence of toxic compounds lignocellulosic hydrolysate.

Yeast cells that grow undergo a mechanism of nutrient transport and assimilation accompanied by the integration of nutrients into cellular components, so that there is an increase in biomass and cell division. The main purpose of growing yeast cells is to multiply cells rather than produce ethanol (Walker & Stewart 2016). However, during fermentation, ethanol production and yeast growth are closely related processes. Therefore, the ability to transport xylose-glucose hydrolysate lignocellulosic is one of the factors that affect the productivity of ethanol production. The genomic recombination of KBKTI 10.5.1 and DBY1 succeeded in improving the genes responsible for xylose-glucose transport to cells, this was indicated by an increase in xylose-glucose consumption in Fcs1 and Fcs4 mutants by 3.64%–4.54% from DBY1 and 1.09%–1.97% from KBKTI 10.5.1 on YPXG media. An increase in xylose-glucose consumption also occurred in both acidic and basic hydrolysate media (Table 4).

## CONCLUSION

Novel strains of Fcs1 and Fcs4 were obtained with different *xyl*1 and *pdc*5 genes from the *Candida tropicalis* KBKTI 10.5.1 and *Saccharomyces cerevisiae* DBY1. The performance of xylose-glucose consumption and ethanol productivity of Fcs1 and Fcs4 mutant on lignocellulosic hydrolysate increased compared to their parents. The ethanol productivity of the Fcs1 mutant increased by 16.67%–27.27%, while the Fcs4 mutant reached 22.22%–33.33% of the parental KBKTI 10.5.1 and DBY1 on acid hydrolysate.

## Figures and Tables

**Table 1:** RAPD primers.

Primers	Sequences (5′-3′)
OPB-12	-CCTTGACGCA-
OPX-03	-TGGCGCAGTG-
OPX-06	-ACGCCAGAGG-
P-20	-AGGAGAACGG-

**Table 2:** Primer pairs of genes that play a role in ethanol metabolism.

Primers	Sequences (5′-3′)	Sources
*xyl*1-F	ATGTCTACTACTCCTACTATTCCTAC	(Kim *et al*. 2019)
*xyl*1-R	TTAAACAAAGATTGGAATGTTGTCCC	
*adh*1-F	AGACGCGCATAACCGCTAGA	(Smidt *et al*. 2012)
*adh*1-R	TAAGATGTGCGCATCTTGGGA	
*xks*1-F	CCAGTGATATCGAGGATGAGATTAGTAC	(Richard *et al*. 2000)
*xks*1-R	CCAGTGATATCTGTACTTGTCAGGGCAT	
*pdc*1-F	ACACCATCTTGGCTTTGGTC	(Kahar *et al*. 2017)
*pdc*1-R	CGAAAGCTGGGAATTGAGTC	
*pdc*5-F	CACGTTGTTGGTGTTCCATC	(Kahar *et al*. 2017)
*pdc*5-R	TCAGTGATCATGGCAGTGGT	
*pdc*6-F	GGAGATTGACCCCAACAAGA	(Kahar *et al*. 2017)

**Table 3:** Test results for parental and mutant yeast ethanol production using xylose and glucose media.

Substrates^a^	Ethanol production performance	Yeast isolate^b^						

		Sc	Ct	Fcs1	Fcs2	Fcs3	Fcs4	Fcs5
Xylose	Sugar consumption (g/L)	0.04	1.94	2.22	1.86	2.52	1.91	2.14
	Ethanol products (g/L)	0.00	0.32	0.32	0.39	0.24	0.39	0.24
	Substrate efficiency (%)	0.19	9.70	11.12	9.31	12.60	9.53	10.62
Glucose	Sugar consumption (g/L)	19.89	19.53	19.65	19.56	19.60	19.56	19.62
	Ethanol products (g/L)	7.34	6.94	6.94	7.02	7.02	7.02	7.02
	Substrate efficiency (%)	99.17	97.67	98.23	97.79	98.02	97.79	98.10

*Notes:*

a The concentration of substrates is 20 g/L fermented for 24 h,

b (Sc) *S. cerevisiae* DBY1, (Ct) *C. tropicalis* KBKTI 10.5.1, (Fcs) mutans strain.

**Table 4:** Parameters of parental and mutant yeast kinetics on different media.

Isolate	Medium^a^	Fermentation time (h)	Percentage of xylose-glucose consumption (%)	Ethanol (g/L)	Yp/s (g/g)	Yx/s (g/g)	Qp (g/L/h)
Sc	YPXG	72	74.90	18.66	0.36	0.04	0.259
	HA	24	30.37	0.79	0.67	0.35	0.033
	HB	24	77.05	0.78	0.61	0.18	0.033

Ct	YPXG	72	76.79	18.08	0.33	0.03	0.251
	HA	24	69.60	0.87	0.31	0.23	0.036
	HB	24	100.00	0.74	0.45	0.25	0.031

Fcs1	YPXG	72	78.30	17.91	0.33	0.03	0.249
	HA	24	69.60	1.00	0.37	0.29	0.042
	HB	24	100.00	0.75	0.47	0.20	0.031

Fcs4	YPXG	72	77.63	17.94	0.33	0.03	0.249
	HA	24	71.76	1.06	0.37	0.29	0.044
	HB	24	100.00	0.75	0.46	0.22	0.031

*Notes:*

a The concentration of xylose-glucose in YPXG media is 20 g/L–50 g/L, acid hydrolysate (HA) media is 3.08 g/L–1.11 g/L and alkaline hydrolysate (HB) is 0.47 g/L–1.30 g/L.

## APPENDIX

### Appendix A Standard Curve

[Table t5-tlsr-34-2-81]

**Table t5-tlsr-34-2-81:**

Standard curve	Concentration (%)	Concentration (g/L)	Area [nRIU*s]	Graph
Ethanol	16		7182290.000	
	8		3624610.000	
	4		1835570.000	
	0.5		206658.000	

Glucose		50	5755390.000	
		40	4684250.000	
		30	3598550.000	
		20	2503300.000	
		10	1528590.000	

Xylose		50	5571150.000	
		40	4499880.000	
		30	3715680.000	
		20	2391470.000	
		10	1366930.000	

Glycerol		2	242931.000	
		1	119783.000	
		0.5	63188.300	
		0.23	29738.200	
		0.1	11820.800	

Lactate	0.125		138300.000	
	0.25		259592.000	
	0.75		648493.000	
	1		953231.000	

Acetate	0.125		112977.000	
	0.25		206550.000	
	0.5		387713.000	
	0.75		527406.000	
	1		688931.000	

### Appendix B Mutant growth on YPG and YPX media

[Table t6-tlsr-34-2-81]

**Table t6-tlsr-34-2-81:**

Wild types and fusant	Media	
	YPG*	YPX*
*Saccharomyces cerevisiae* (DBY1)	No Dilution	No Dilution
*Candida tropicalis* (KBKTI 10-5.1)	No Dilution	No Dilution
Mutant (Fcs)	No Dilution	Dilution 1:10

*Note*:

* Yeast cultures are spread as much as 100 L and incubated for 3 days at 30ºC.

### Appendix C Concentration of xylose and glucose elephant grass hydrolysate

[Table t7-tlsr-34-2-81]

**Table t7-tlsr-34-2-81:**

	Acid hydrolysate				Alkaline hydrolysate			

	1%		3%		1%		3%	

	Xylose (g/L)	Glucose (g/L)	Xylose (g/L)	Glucose (g/L)	Xylose (g/L)	Glucose (g/L)	Xylose (g/L)	Glucose (g/L)
Cellulose of elephant grass (1 month old)	3.03	0.95	3.08	1.11	0.59	1.06	0.47	1.30

### Appendix D Performance of ethanol production the parental and mutant yeast on YPXG media

[Table t8-tlsr-34-2-81]

**Table t8-tlsr-34-2-81:**

Isolate	Ethanol (%)				Glucose (g/L)				Xylose (g/L)				Glycerol(g/L)				Lactate (g/L)				Acetate (g/L)				OD600			

	Fermentatlor 1 Time (h)																											

	0	24	48	72	0	24	48	72	0	24	48	72	0	24	48	72	0	24	48	72	0	24	48	72	0	24	48	72
**Sc**	0.0	2.31	2.36	2.37	50	0.00	0.01	0.02	20	18.47	17.57	17.55	0.0	1.81	1.68	1.76	0.0	0.81	1.02	1.16	0.0	0.00	0.07	0.06	0.4	3.40	4.70	3.80
**Ct**	0.0	2.24	2.24	2.29	50	0.02	0.01	0.01	20	17.00	16.29	16.23	0.0	1.47	1.48	1.38	0.0	3.18	3.58	3.38	0.0	0.06	0.06	0.01	0.4	3.90	4.70	4.37
**Fcs1**	0.0	2.15	2.27	2.27	50	1.47	0.02	0.02	20	16.62	16.12	15.18	0.0	1.60	1.68	1.61	0.0	2.80	4.41	5.15	0.0	0.05	0.10	0.11	0.4	3.37	4.80	4.00
**Fcs4**	0.0	2.20	2.22	2.27	50	0.00	0.02	0.02	20	16.69	16.16	15.64	0.0	1.30	1.41	1.36	0.0	2.77	3.63	3.20	0.0	0.12	0.11	0.09	0.4	3.87	5.20	4.80

### Appendix E Performance of ethanol production the parental and mutant yeast on acid hydrolysate

[Table t9-tlsr-34-2-81]

**Table t9-tlsr-34-2-81:**

Isolate	Ethanol (%)				Glucose (g/L)				Xylose (g/L)				Glycerol (g/L)				Lactate (g/L)				Acetate (g/L)				OD600			

	Fermentation Time (h)																											

	0	4	8	24	0	4	8	24	0	4	8	24	0	4	8	24	0	4	8	24	0	4	8	24	0	4	8	24
**Sc**	0.0	0.74	0.74	0.79	1.11	0.19	0.00	0.00	3.08	2.99	2.98	2.92	0.0	0.30	0.35	0.37	0.0	0.02	0.01	0.01	0.0	0.00	0.00	0.00	0.37	0.60	1.00	1.20
**Ct**	0.0	0.86	0.86	0.87	1.11	0.01	0.00	0.00	3.08	2.41	1.74	1.27	0.0	0.07	0.09	0.25	0.0	0.02	0.01	0.11	0.0	0.00	0.00	0.00	0.50	0.69	1.63	2.16
**Fcs1**	0.0	0.94	0.98	1.00	1.11	0.02	0.00	0.00	3.08	2.41	2.07	1.27	0.0	0.08	0.11	0.31	0.0	0.02	0.01	0.11	0.0	0.00	0.00	0.00	0.46	0.5	1.49	2.57
**Fcs4**	0.0	0.95	1.04	1.06	1.11	0.01	0.00	0.00	3.08	2.47	2.13	1.18	0.0	0.11	0.12	0.29	0.0	0.02	0.01	0.2	0.0	0.00	0.00	0.00	0.48	0.77	1.57	2.60

### Appendix F Performance of ethanol production the parental and mutant yeast on alkaline hydrolysate

[Table t10-tlsr-34-2-81]

**Table t10-tlsr-34-2-81:**

Isolate	Ethanol (%)				Glucose (g/L)				Xylose (g/L)				Glycerol (g/L)				Lactate (g/L)				Acetate (g/L)				OD600			

	Fermentation Time (h)																											

	0	4	8	24	0	4	8	24	0	4	8	24	0	4	8	24	0	4	8	24	0	4	8	24	0	4	8	24
**Sc**	0.0	0.76	0.76	0.78	1.30	0.15	0.14	0.00	0.47	0.47	0.44	0.41	0.0	0.31	0.32	0.35	0.0	0.02	0.02	0.03	0.0	0.00	0.00	0.00	0.37	0.39	0.74	0.89
**Ct**	0.0	0.69	0.74	0.74	1.30	0.18	0.14	0.00	0.47	0.33	0.18	0.00	0.0	0.24	0.26	0.04	0.0	0.01	0.02	0.02	0.0	0.00	0.00	0.00	0.50	0.74	0.82	1.51
**Fcs1**	0.0	0.74	0.75	0.75	1.30	0.19	0.17	0.00	0.47	0.33	0.28	0.00	0.0	0.26	0.27	0.06	0.0	0.01	0.02	0.02	0.0	0.00	0.00	0.00	0.46	0.46	0.97	1.78
**Fcs4**	0.0	0.74	0.74	0.75	1.30	0.14	0.10	0.00	0.47	0.30	0.18	0.00	0.0	0.26	0.27	0.07	0.0	0.01	0.02	0.03	0.0	0.00	0.00	0.00	0.48	0.54	0.92	1.73
